# Cardiorespiratory, Sedative and Antinociceptive Effects of a Medetomidine Constant Rate Infusion with Morphine, Ketamine or Both

**DOI:** 10.3390/ani11072081

**Published:** 2021-07-13

**Authors:** Lucas Troya-Portillo, Javier López-Sanromán, María Villalba-Orero, Isabel Santiago-Llorente

**Affiliations:** 1 Departamento de Medicina y Cirugía Animal, Universidad Complutense de Madrid, 28040 Madrid, Spain; lsroman@ucm.es (J.L.-S.); mvorero@gmail.com (M.V.-O.); 2 Hospital Clínico Veterinario Complutense, Universidad Complutense de Madrid, 28040 Madrid, Spain; isabel.santiago3@gmail.com

**Keywords:** horse, alpha-2-agonist, opioids, mechanical stimulus

## Abstract

**Simple Summary:**

Standing surgery and diagnostic procedures in equine patients under deep sedation reduce the risk associated with general anesthesia. Sedation protocols must be safe, provide a good quality of sedation without producing cardiorespiratory depression and severe ataxia. The use of adrenergic alpha-2 receptors agonist in combination with opioids and/or ketamine can achieve an adequate sedation and provide sufficient analgesia for surgical procedures. Medetomidine and medetomidine with morphine in intravenous constant rate infusion have been evaluated for standing sedation but have not been compared directly. Although ketamine has been combined with other alpha-2 agonists successfully, it has not been evaluated in combination with medetomidine. The objective of this study was to compare four medetomidine-based protocols with the addition of morphine and/or ketamine, including cardiorespiratory, sedative and mechanical antinociceptive variables. All four protocols produced a similar degree of sedation and mechanical antinociception without clinically relevant impact on cardiorespiratory variables.

**Abstract:**

Standing surgery under sedation reduces anesthetic-related mortality in horses. Medetomidine, alone and combined with morphine in a constant rate infusion (CRI), has been described for standing surgery but their cardiorespiratory, sedative and antinociceptive effects have never been compared. The addition of ketamine could improve analgesia in these procedures with minimal cardiorespiratory consequences. The objectives were to compare the cardiorespiratory effects, quality of sedation, antinociception and ataxia produced by administration of a medetomidine-based CRI with morphine, ketamine or both, in standing horses. A prospective, blind, randomized crossover, experimental design with six healthy adult horses was performed, in which four treatments were administered to all horses with at least two weeks of washout period: medetomidine (M); medetomidine and ketamine (MK); medetomidine and morphine (MMo); and medetomidine, morphine and ketamine (MMoK). Dosages were the same in all treatment groups: medetomidine at 5 µg/kg bwt followed by 5 µg/kg bwt/h, ketamine at 0.4 mg/kg/h and morphine at 50 µg/kg bwt, followed by morphine 30 µg/kg bwt/h. Drug infusions were maintained for 120 min. Cardiorespiratory variables, sedation degree and antinociceptive effects were evaluated during the procedure. All combinations produced similar sedation and antinociceptive effects and no clinically relevant alterations in cardiorespiratory variables occurred. Medetomidine CRI combined with morphine, ketamine or both are suitable and safe protocols for standing sedation in horses and the addition of morphine and/or ketamine did not cause any negative effect but no improving effect on sedation and antinociception was detected.

## 1. Introduction

General anesthesia-related mortality is higher in horses compared to small animals or humans [1,2]. Cardiac arrest, fractures and myopathies, which are mainly associated with cardiorespiratory depression caused by inhalant agents and traumatic complications during induction and recovery are the main factors leading to mortality [3,4,5,6]. Standing sedation decreases the risk associated with general anesthesia while allowing the performance of several surgeries [7,8]. Eligible drugs for standing procedures must provide adequate sedation and analgesia, producing minimal ataxia and cardiopulmonary depression. These drugs are usually administered in a constant rate infusion (CRI), as this offers more stable sedation and reduces adverse effects, compared with repeated bolus administration [8]. 

The combination of drugs with different mechanisms of action, multimodal sedation and analgesia, reduces adverse effects and enhances the desired effects [9]. Medetomidine possesses a great affinity for the α-2 adrenergic receptor; thus, inducing a high level of sedation and antinociception. Due to its short half-life, medetomidine is an ideal candidate for CRI administration [10]. Similar to other α-2 adrenergic receptor agonists, this drug causes dose dependent cardiovascular depression, ataxia and a decrease in intestinal motility [11,12]. Medetomidine CRI alone has been positively experimentally evaluated for long-term sedation although further studies evaluating the analgesic properties are necessary [10]. Morphine is a full opioid receptor agonist with analgesic effects, although, at high doses it increases locomotor activity, induces respiratory depression and intestinal hypomotility [13,14,15,16,17,18]. The combination of morphine with medetomidine has been shown to provide adequate sedation for laparoscopy without concerning cardiorespiratory adverse effects, as well as its combination with other α-2 adrenergic receptor agonists [19,20]. Ketamine is an antagonist of the N-methyl-d-aspartate (NMDA) receptors commonly used in equine anesthesia and, at low doses, presents anti-inflammatory and potent analgesic effects [21,22]. Ketamine, used in combination with α-2 adrenergic receptor agonists and/or opioids, provides somatic analgesia and a rapid onset of sedation with minimal adverse cardiorespiratory effects, although it can produce ataxia and increased locomotor activity by increasing skeletal muscle tone [21,22,23]. Ketamine in addition to an α-2 adrenergic receptor agonist based CRI has been shown to improve sedation quality [24].

The combination of medetomidine and morphine has previously been described for standing laparoscopy, but the effects of adding morphine was not described [19]. Our hypothesis was that the addition of ketamine and/or morphine to a medetomidine CRI might improve sedative and analgesic properties, without a significant negative impact on cardiorespiratory variables, but it has not been investigated directly. To our knowledge, this is the first report that combines medetomidine, medetomidine–ketamine, medetomidine–morphine and medetomidine–ketamine–morphine in a CRI and compares the cardiorespiratory effects, degree of sedation and antinociception produced in horses. 

## 2. Materials and Methods

### 2.1. Study Design

A prospective blind randomized crossover study was designed. Four sedation protocols were used in each horse, with a two-week washout period between treatments. This study was approved by the Institutional Animal Care Committee (Madrid, Spain; ES280790000101).

### 2.2. Animals

Six mature crossbred horses (three mares and three geldings) from the teaching herd, with a mean ± SD (standard deviation) age of 11 ± 3 years (range, 7–14 years) and a mean weight (BW) of 472 ± 52 kg (range, 390–565 kg) were included. All horses were considered healthy following a routine physical examination. The sample size was estimated using the sedative (height of head above the ground [HHAG]) and antinociceptive (mechanical) results of a pilot study and data obtained in other studies with similar methodology. An HHAG mean of 50 ± 10% and a nociceptive threshold of 20 ± 12 N. Accepting an alpha risk of 0.05 and a beta risk of 0.2, 6 subjects were necessary in each group to recognize as statistically significant a minimum difference of 0.2 units between any pair of groups with 4 groups.

### 2.3. Sedation Protocols

Horses received a CRI of medetomidine (M), medetomidine–ketamine (MK), medetomidine–morphine (MMo) and medetomidine–ketamine–morphine (MMoK) for two hours (Table 1). Infusion solutions were prepared by adding 5 mL of medetomidine (Sedastart^®^ 1 mg/mL, B. Braun VetCare SA, Barcelone, Spain) in all treatment groups, 4 mL of ketamine (Ketamidor^®^ 100 mg/mL, Richter Pharma AG, Wels, Austria) in MK and MMoK treatment groups, and 1.5 mL of morphine (Morfina B. Braun 2%^®^, B. Braun Medical, Barcelone, Spain) in MMo and MMoK treatment groups to a 500 mL saline solution bottle (NaCl 0.9%, B. Braun, B. Braun VetCare SA, Barcelone, Spain). Before the addition of the drugs, the same volume of saline solution was discarded to have a final volume of 500 mL. All solutions were administered by an electronic infusion pump at 0.5 mL/kg/h (Infusomat^®^, B. Braun Surgical, Barcelone, Spain). The order of the procedures was established randomly using computer software.

### 2.4. Experimental Procedure

All horses were fasted for 12 h before the experiment, although free access to water was allowed until the beginning of the procedure. Prior to any drug administration, horses were placed inside a stock in a quiet closed room. A 14G intravenous catheter (Surflo^®^ 14G 64 mm, Terumo Europe NV, Leuven, Belgium) in the left jugular vein and a 20G (Surflo^®^ 20G 32 mm, Terumo Europe NV, Leuven, Belgium) catheter in the left facial or facial transverse artery were placed with an aseptic technique using subcutaneous administration of mepivacaine (Mepivacaina B. Braun 20 mg/mL, B.Braun Medical S.A, Barcelone, Spain). Once basal variables were registered, bolus 1 was administered (time −10). After 10 min (time 0) bolus 2 was administered (bolus 2) and the respective CRI was infused and maintained for 120 min. All horses were observed for 48 h after the procedure by the equine hospitalization veterinary team and significant complications, such as abdominal discomfort or abnormal physical exam variables, were registered.

### 2.5. Cardiorespiratory Monitoring

Heart rhythm and heart rate (HR) was acquired using a telemetric Holter device (Televet^®^ 100, Engel Engineering Service GmbH, Heusenstamm, Germany) with electrodes in a base–apex lead, recording an electrocardiogram continuously for at least five minutes prior to administration of any drug until the end of the CRI. The onset of cardiac arrhythmias was reported and HR was registered at baseline and every 10 min. In addition, PR, QT and corrected QT (QTc = QT/HR) intervals and QRS complex were measured in three sinusal consecutive heartbeats and expressed as the mean ± SD at baseline, prior to any drug administration, after the medetomidine bolus (−5), at 60 and 120 min. Invasive systolic, mean and diastolic blood pressure (SAP, MAP and DAP, respectively) were registered at baseline and every 10 min. Measurements were obtained by connecting the arterial catheter to a pressure transducer setting the zero point reference at the level of the right atrium and connected to a multiparameter monitor (VetCare^®^, B. Braun VetCare SA, Barcelone, Spain). Cardiac output (CO) was obtained with pulsed-wave Doppler of the pulmonary artery as previously described and calculated with the image analysis software of the echocardiograph (Esaote^®^ MyLab30Vet, Esaote S.p.A., Florence, Italy) [25]. For this calculation, a two-dimensional echocardiography of the right ventricle outflow tract was obtained and the diameter of the pulmonary valve was measured. The velocity time integral (VTI) was acquired using pulsed-wave Doppler, by placing the cursor immediately after the pulmonary valve, aligning the ultrasound beam as parallel with flow as possible. The VTI was traced with electronic calipers and reported as area under the curve (cm^2^). HR was calculated by measuring the RR interval preceding the traced VTI. Cardiac index (CI_bw_ = CO/bodyweight) was then calculated and expressed as the percentage of variation compared with baseline values. Echocardiography was blindly performed and images were registered at baseline and at 30, 60, 90 and 120 min during the infusion in each treatment group. Analysis of the images to obtain CO was done offline by the same operator that performed the echocardiography, which was also blinded during the analysis. 

Respiratory rate was assessed by observation of the thoracic movements over 30 s and registered at baseline and every 10 min. PaO_2_, PaCO_2_, pH, plasmatic electrolyte concentrations (Na^+^, K^+^, iCa, HCO_3_^−^), standard base excess and oxygen saturation (SO_2_) were obtained by processing an arterial blood sample obtained anaerobically from the arterial catheter with a blood gas analyzer (IRMA Trupoint^®^, Lifehealth, Minneapolis, MN, USA). Samples were obtained prior to any drug administration (baseline) and at 30, 60 and 120 min during the CRI.

### 2.6. Sedation Assessment

HHAG was used for sedation degree evaluation as previously described [26,27]. A modified technique to that described by Ringer et al. was used, in which the distance in centimeters to the ground was taken from the lowest point of the lip prior to the administration of any drug and every 10 min [27]. All measurements were expressed as an increased or decreased percentage of the initial measurement and a decrease ≥ 50% was considered as adequate sedation, as described elsewhere [26,27]. In addition, ataxia was assessed by two blinded evaluators with extensive experience in equine anesthesia and expressed using a visual analogic scale (VAS) consisting of a 10 cm line, where zero represented a normal horse and 10 a severely ataxic horse unable to stand. 

### 2.7. Antinociceptive Effect

Mechanical nociception stimulation was performed by two trained blinded evaluators with a hand-held portable algometer, provided with a 1.5 mm diameter blunt ended probe, ranking from 10 to 100 N (Wagner FPN 100^®^, Wagner Instruments, Greenwich, CT, USA) as described elsewhere [28,29,30,31]. Each evaluator was assigned a forelimb during all procedures and two measurements were performed per assessor at each site prior to administration of any drug and every 20 min during sedation, applying a constantly increasing pressure perpendicular to the skin in three anatomical areas (half of the dorsal side of the cannon bone, coronary band and lateral heel) in a random order, until a response was obtained from the horse (withdrawal reflex) or until it reached a maximum of 40 N. The test areas were clipped to have direct contact to the skin and to assure the same point was tested at each time. Before and after the procedure all test areas were inspected to ensure that there was no skin or soft tissue damage. The maximum force applied was registered by the algometer, and the final value consisted of the median of the four values (two per evaluator and per location). If the endpoint (40 N) was reached, this value was recorded.

### 2.8. Data Analysis

Data analysis was performed using statistical software (GraphPad Prism 7.00, GraphPad Software, San Diego, CA, USA). A Kolmogorov–Smirnov test was first performed to test normality. Numerical variables were expressed as mean ± SD or mean (range) for normally and non-normally distributed data, respectively. A two-way analysis of variance (ANOVA) with an inter-subject factor was performed. Post hoc comparisons between each time and treatment group were performed using a Tukey post-test. A Chi-Squared Test was performed to compare the incidence of arrhythmias between protocols. *p* values < 0.05 were considered significant. 

## 3. Results

All horses included completed the study. No complications were observed during the procedure nor in the following 48 h. 

### 3.1. Cardiovascular and Respiratory Variables

No differences between treatment groups regarding HR, SAP, MAP, DAP, CI_bw_, PR intervals, QRS complex and QTc were found and all maintained these variables at physiological levels (Table 2, Tables S1 and S2). Horses receiving MMo and MK had an increased QTc compared with baseline although no differences between treatments were found. In all treatment groups, horses presented frequent sinoatrial and second-degree atrioventricular blocks after the medetomidine bolus administration. These bradyarrhythmias were maintained during almost all the procedure in some horses (M: 2/6; MK: 5/6; MMo: 3/6; MMoK: 4/6) without differences between protocols. One horse showed occasional supraventricular premature complexes in three of the procedures (M, MK, MMoK). Additionally, in these same treatment groups, another horse developed an occasional supraventricular premature complex, although it was a different horse in each procedure.

Horses included in MK or MMo groups had a higher RR before drug administration (Table 2). In all protocols, the RR decreased after the bolus, but no differences between treatment groups were found. The mean PaCO_2_ was above the reference range at 120 min in all treatment groups but were not significantly different between protocols. The pH in the MMo group was above the reference range from minute 60. The plasmatic electrolyte concentration did not show any relevant change (Table S3).

### 3.2. Sedative Effects

**Figure 1:**
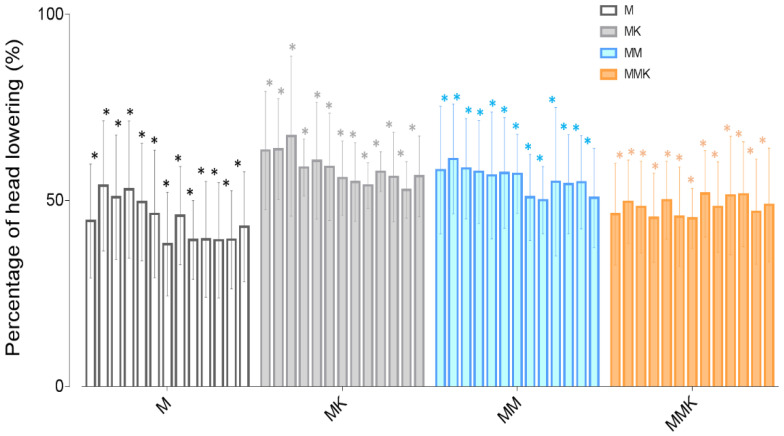
Percentage of head height to ground during the infusion every 10 min, with baseline as reference (100%) for sedation assessment. * Significantly different (*p* < 0.05) from baseline within a treatment.

In all studied protocols, HHAG decreased over time but no differences were observed between protocols (Figure 1 and Table S4). Overall, no significant differences were observed concerning the ataxia degree (Table S5). Ataxia after the medetomidine bolus was higher in the MK group but became similar to the other treatment groups after 10 min. 

### 3.3. Antinociceptive Effect

Mechanical nociception threshold (MNT), measured at the coronary band, heels and cannon bone, was higher when compared to the onset of the procedure. All three locations had a similar tendency (Table 3).

## 4. Discussion

This study reveals that the addition of ketamine, morphine or ketamine–morphine to a medetomidine CRI in horses, while maintaining clinically acceptable cardiopulmonary variables, does not provide additional sedation and antinociceptive effects based on the mechanical nociception stimulation used in this study. Although adverse effects related to the administration of these drugs, such as behavioral changes and decreases in intestinal motility [15,17,32,33], have not been directly evaluated in this study, no complications were observed during the procedure or the following 48 h.

The four standing sedative protocols studied showed similar cardiorespiratory effects with no clinically relevant differences at any time. Administration of medetomidine triggered sinoatrial and second-degree atrioventricular blocks. The administration of α-2 adrenergic receptor agonists cause an initial increase in systemic vascular resistance, producing hypertension and a baroreceptor-mediated reflex bradycardia, followed by a centrally mediated hypotension [34,35,36]. In our study, no difference in MAP and CI was observed in any of the procedures. In contrast with our study a significant decrease of CI_bw_ has been described during 50 min after a bolus administration of 5 µg/kg of intravenous medetomidine [12]. Similarly to our results a combination of medetomidine and morphine in CRI during standing laparoscopy did not cause a significant decrease in cardiovascular variables [19]. Cardiovascular adverse effects are influenced by dose and speed of administration, which could explain why fewer and less severe adverse effects are seen when medetomidine is administered as a CRI [8,10,34] in comparison with bolus administration [12]. In our study we did not observe differences regarding the cardiovascular variables between protocols, we did not see any cardiovascular adverse effect and all values were within acceptable ranges, supporting that medetomidine in a CRI alone or combined with ketamine and morphine, at the dose used in our study, does not produce a clinically relevant effect in cardiovascular variables. Although measuring at the level of the aorta is the gold standard for arterial blood pressure, the use of a peripheral artery is more practical and is performed routinely in the daily clinic [37]. The facial artery was chosen because is easily palpable and accessible. Head height decrease has been described to decrease arterial blood pressure measurements in standing horses [38]. Thus, due to the head position, blood pressure cannot be accurately described in this report. However, head height lowering was similar between groups and; therefore, the result observed can be attributed to the treatment groups. The use of doppler echocardiography to estimate cardiac output is widely used and presents good correlation with other invasive techniques [39]. However, absolute values obtained may not represent real CO and thus CI_bw._ However, echocardiography is useful to monitor hemodynamic changes over time and in response to treatments with the advantage of being a non-invasive technique [40]. Although RR was decreased with all protocols, PaO_2_ and PaCO_2_ did not differ throughout the procedures. Bradypnea and mild hypercapnia have been described after both medetomidine and morphine administration [12,19], although, in accordance with other authors, the combination of both did not enhance respiratory depression in our study [41,42]. 

In all the combinations studied, the sedation degree was adequate and there were no differences between protocols, which may indicate that the addition of morphine or ketamine does not enhance sedation. These results are similar to other studies, where the addition of butorphanol or methadone did not produce a decrease in the ground-to-lip distance in comparison with the administration of α-2 adrenergic receptor agonists alone [26,27,43,44]. In addition, ketamine has a lack of sedative effect and; therefore, an increase in sedation was not expected [38]. In horses, α-2 adrenergic receptor agonists alone are able to achieve deep plane sedations and we may not be able to evaluate the potential increase produced by the combinations. HHAG is an objective measure that has been largely use to evaluate sedation [45]. Recently, a sedation scale in horses has been described and compared to other methods [46]; however, this scale required interaction with the horse and may alter results. Combination of medetomidine with other drugs such as opioids may allow decreasing both drug doses to achieve the same grade of sedation, although further studies are warranted to describe the optimal dosage. 

In our study, the mechanical nociceptive threshold was similar in all protocols. This result suggests that the antinociception induced by medetomidine is not enhanced by the addition of other analgesic drugs, such as ketamine and/or morphine. As it is well known, α-2 adrenergic receptor agonists increase the nociceptive threshold in horses and donkeys [47,48,49,50]. In contrast, systemic opioids do not have such an evident effect on nociceptive thresholds and several studies show confusing results. An increase in the nociceptive threshold with butorphanol, levomethadone or with both combined with α-2 adrenergic receptor agonists has been reported [44,51,52,53,54,55,56]. On the contrary, some studies with butorphanol or fentanyl have not evidenced an increase in nociceptive thresholds [57,58]. Moreover, the analgesic efficacy of morphine is inconsistent both in awake and anaesthetized horses, which could explain our results [56,59]. It should be noted that in our study healthy pain-free horses were used, similarly to other opioid studies, and analgesic efficacy of morphine has been proven in horses that are in pain, and so could be beneficial in a clinical setting in the treatment of perioperative pain [60]. Although we did not find an additional analgesic effect when ketamine was used, this drug has previously shown a significant increase in thermal and electrical nociceptive threshold with local and systemic administration and a synergistic effect with α-2 adrenergic receptor agonists [7,22,61,62]. Another explanation for the absence of differences between protocols could be that the nociceptive threshold achieved with medetomidine alone reached our security limit, which may hamper assessing differences in nociception above this limit caused by morphine and/or ketamine. However, the cut-off value used in this study is similar to that used in similar studies [42,44]. Mechanical nociception threshold with pressure algometry stimulates both Aδ and C nerve nociceptive fibers [63], and has been widely use in equine research and clinical setting [31]. Electrical and thermal nociceptive threshold have been also evaluated in horse. Mechanical stimulus was chosen in our study because it have shown to be more sensitive [64,65] and has a lower risk of lesions in the animal. Although no differences in antinociception could be noted in our study with the addition of morphine and/or ketamine, it cannot be excluded differences in nociceptive values above our security limit (40 N). 

Excessive ataxia during standing surgery can hinder the procedure, increasing surgical time and the risk of surgical complications. No considerable ataxia was found in our study and no differences between treatment groups were evident. Ataxia is a common effect after α-2 adrenergic receptor agonist administration and it has been described as being greater when combined with butorphanol [26,27]. Morphine has been described as causing less ataxia than other opioids and could explain the low ataxia shown in this report [66]. In another study, the addition of a higher dose of ketamine that such used in this report to a romifidine CRI did not increase the ataxia score, similarly to our results [24].

Cautious extrapolation to the clinical scenario must be done. Several limitations must be pointed out from our study. Firstly, although sample size was calculated to detect differences in sedation and mechanical nociceptive threshold, a small sample size may have hampered to highlight slights changes between treatment groups. In horses there is not a validated method to accurately evaluate sedation. In this report, HHAG was used to assess sedation because it is widely used and described making it a standardized and feasible method to compare between studies [26,27,67]. As previously discussed mechanical nociception have been extensively used to evaluate analgesic protocols [68]. Although useful to assess nociception, this type of stimulus may not represent surgical pain [69], and mechanical nociceptive threshold has an intrinsic limitation due to the activation of not only nociceptors but mechanoceptors that can elicit an early response to pressure [68]. Therefore, a clinical study may be necessary to extrapolate the nociceptive results obtained in this report to the clinical settings. 

## 5. Conclusions

In conclusion, CRI administration of medetomidine combined with morphine, ketamine or both produced an adequate degree of sedation and antinociception, without clinically relevant changes in cardiorespiratory variables and without the appearance of complications. In our study, the addition of morphine and/or ketamine did not produce any negative effect in cardiorespiratory, sedative and ataxia variables.

## Figures and Tables

**Table 1:** Bolus and CRI dosages of the four sedation protocols. M (medetomidine), K (ketamine), Mo (morphine), ml (milliliter), mg (milligram), µg (microgram), h (hour).

Treatment Group	Drug	Bolus 1	Bolus 2	CRI Dosage
M	Medetomidine	5 µg/kg bwt	-	5 µg/kg bwt/h
	Saline 0.9%	-	5 mL	
MK	Medetomidine	5 µg/kg bwt	-	5 µg/kg bwt/h
	Ketamine	-	-	0.4 mg/kg bwt/h
	Saline 0.9%	-	5 mL	
MMo	Medetomidine	5 µg/kg bwt	-	5 µg/kg bwt/h
	Morphine	-	50 µg/kg bwt	30 µg/kg bwt/h
	Saline 0.9%	-	Up to 5 mL	
MMoK	Medetomidine	5 µg/kg bwt		5 µg/kg bwt/h
	Ketamine	-	-	0.4 mg/kg bwt/h
	Morphine	-	50 µg/kgbwt	30 µg/kg bwt/h
	Saline 0.9%	-	Up to 5 mL	

**Table 2:** Heart rate (HR), respiratory rate (RR), mean arterial pressure (MAP) and cardiac index (CI) before administration of any drug (baseline), and during the infusion in the four treatment groups. * Significantly different (*p* < 0.05) from baseline within a treatment. ** Significantly different (*p* < 0.05) from time 0 within a treatment ^a,b,c,d^ Different superscript letters indicate significant differences (*p* < 0.05) between treatments at this timepoint.

	Baseline	0	10	20	30	40	50	60	70	80	90	100	110	120
HR (bpm) Reference range: 24–44 bpm														
M	37 ± 4	30 ± 7	30 ± 7	31 ± 5	32 ± 5	31 ± 8	33 ± 6	32 ± 6	30 ± 5	30 ± 5	30 ± 5	30 ± 4	31 ± 6	31 ± 6
MK	45 ± 11	32 ± 6 *	33 ± 7 *	35 ± 7 *	34 ± 8 *	33 ± 7 *	34 ± 6 *	34 ± 6 *	32 ± 7 *	32 ± 7 *	32 ± 6 *	32 ± 7 *	34 ± 8 *	32 ± 5 *
MMo	40 ± 7	30 ± 5 *	31 ± 6 *	31 ± 8 *	34 ± 9	31 ± 6 *	32 ± 7	30 ± 3 *	33 ± 6	32 ± 6	33 ± 5	35 ± 8	32 ± 7	33 ± 5
MMoK	38 ± 4	30 ± 4	29 ± 5	29 ± 6 *	31 ± 7	30 ± 5	31 ± 7	31 ± 6	32 ± 8	33 ± 8	32 ± 6	31 ± 4	35 ± 6	32 ± 4
RR (rpm) Reference range: 10–24 bpm														
M	14 ± 5 ^a,b^	8 ± 2 *	8 ± 1 *	8 ± 2 *	8 ± 1 *	7 ± 1 *	7 ± 2 *	7 ± 2 *	7 ± 2 *	6 ± 2 *	7 ± 2 *	7 ± 1 *	7 ± 2 *	7 ± 1 *
MK	27 ± 7 ^a,c,d^	12 ± 3 *	11 ± 3 *	10 ± 3 *	11 ± 3 *	8 ± 2 *	9 ± 3 *	9 ± 2 *	8 ± 5 *	10 ± 4 *	8 ± 2 *	7 ± 3 *^,^**	8 ± 3 *	7 ± 3 *^,^**
MMo	21 ± 6 ^b,c^	12 ± 4 *	10 ± 3 *	9 ± 4 *	9 ± 3 *	7 ± 3 *	8 ± 3 *	7 ± 3 *	7 ± 3 *	8 ± 4 *	6 ± 3 *^,^**	7 ± 3 *	7 ± 3 *^,^**	7 ± 2 *
MMoK	18 ± 6 ^d^	11 ± 3 *	7 ± 1 *	6 ± 1 *^,^**	6 ± 1 *^,^**	6 ± 2 *	6 ± 1 *^,^**	6 ± 1 *^,^**	7 ± 2 *	6 ± 1 *^,^**	6 ± 1 *^,^**	6 ± 3 *^,^**	6 ± 1 *^,^**	6 ± 1 *^,^**
MAP (mmHg) Reference range: 100–120 mmHg														
M	116 ± 16	109 ± 18	102 ± 19	99 ± 15	105 ± 14	104 ± 14	95 ± 14	94 ± 15	98 ± 14	95 ± 15	96 ± 14	96 ± 13	97 ± 15	94 ± 11
MK	127 ± 29	125 ± 26	116 ± 22	116 ± 21	112 ± 23	107 ± 17	105 ± 18	100 ± 14 *	102 ± 15	106 ± 23	107 ± 20	110 ± 21	104 ± 26	113 ± 29
MMo	124 ± 31	119 ± 21	109 ± 18	108 ± 13	107 ± 13	104 ± 13	104 ± 16	106 ± 17	100 ± 16	98 ± 14	100 ± 16	100 ± 11	102 ± 11	101 ± 12
MMoK	118 ± 20	116 ± 13	106 ± 10	103 ± 12	100 ± 14	102 ± 15	98 ± 18	98 ± 20	98 ± 19	98 ± 20	95 ± 16	96 ± 19	97 ± 19	99 ± 16
CI (% of change from baseline)														
M					−23.3 ± 14.7			−13.4 ± 33.9			−23.4 ± 24.8			−20.5 ± 16
MK					−12.6 ± 19.2			−29.5 ± 14.2			−22.2 ± 30.2			−29.8 ± 15.5
MMo					−27.6 ± 27.8			−33.0 ± 30.7			−27.3 ± 39.7			−34.1 ± 28.4
MMoK					−21.0 ± 20.8			−19.8 ± 26.2			−22.9 ± 20.5			−9.88 ± 23.2

**Table 3:** Mechanical nociception: Maximum pressure (N) applied with the manual algometer at each anatomic site, before any drug administration (baseline) and each 20 min during the infusion in the four treatment groups, with a security endpoint of 40 N. * Significantly different (*p* < 0.05) from baseline within a treatment. ^a^ Different superscript letters indicate significant differences (*p* < 0.05) between treatments at this timepoint. ** Significantly different (*p* < 0.05) from time 0 within a treatment.

	Baseline	0	20	40	60	80	100	120
Heel								
M	28 ± 7	40 ± 0 *	39 ± 2 *	40 ± 1 *	40 ± 0 *	40 ± 0 *	40 ± 0 *	40 ± 0 *
MK	26 ± 6	37 ± 5 *	37 ± 5 *	36 ± 7 *	39 ± 6 *	38 ± 8 *	38 ± 8 *	38 ± 8 *
MMo	25 ± 7	37 ± 5 *	37 ± 5 *	36 ± 5 *	39 ± 2 *	38 ± 6 *	38 ± 6 *	38 ± 5 *
MMoK	27 ± 10	34 ± 7	37 ± 5 *	37 ± 6 *	38 ± 3 *	40 ± 1 *	40 ± 0 *	40 ± 0 *
Cannon bone								
M	24 ± 9	40 ± 0 *^,a^	39 ± 2 *	38 ± 4 *	40 ± 1 *	39 ± 3 *	40 ± 0 *	38 ± 4 *
MK	29 ± 7	37 ± 6 *	33 ± 10	35 ± 9	38 ± 5 *	35 ± 9	36 ± 8	37 ± 7 *
MMo	26 ± 10	39 ± 2 *	39 ± 2 *	40 ± 0 *	40 ± 0 *	40 ± 0 *	37 ± 4 *	40 ± 0 *
MMoK	24 ± 10	32 ± 10 ^a^	38 ± 5 *	38 ± 5 *	39 ± 3 *	39 ± 2 *	40 ± 0 *^,^**	40 ± 0 *^,^**
Coronary band								
M	30 ± 7	40 ± 0 *^,a^	40 ± 1 *	40 ± 1 *	40 ± 0 *	39 ± 2 *	40 ± 0 *	40 ± 0 *
MK	26 ± 10	35 ± 7 *^,a^	36 ± 6 *	36 ± 6 *	37 ± 5 *	36 ± 7 *	35 ± 7 *	37 ± 6 *
MMo	28 ± 8	38 ± 4 *	40 ± 1 *	39 ± 2 *	40 ± 1 *	40 ± 0 *	40 ± 0 *	40 ± 0 *
MMoK	29 ± 10	36 ± 5 *	37 ± 5 *	38 ± 3 *	39 ± 3 *	40 ± 1 *	40 ± 0 *	40 ± 0 *

## Data Availability

The data presented in this study are available on request from the corresponding author. The data are not publicly available due to privacy policies.

## Supplementary Materials

The following are available online at https://www.mdpi.com/article/10.3390/ani11072081/s1, Table S1. Doppler echocardiographic measurement of cardiac output at the pulmonary artery. Table S2. Electrocardiographic intervals duration. Table S3. Partial gas arterial pressures, electrolytes and acid-base parameters. Table S4. Percentage of head height to ground with baseline as reference (100%). Table S5. Visual Analogic Scale evaluation of ataxia (cm) between 0 (normal horse) to 10 (horse unable to stand).
