# Supplementary material for: Cardiorespiratory, Sedative and Antinociceptive Effects of a Medetomidine Constant Rate Infusion with Morphine, Ketamine or Both
Source: Animals (Basel). 2021 Jul 13;11(7):2081. doi: 10.3390/ani11072081 (PMC8300393; doi:10.3390/ani11072081)
Supplement: Supplementary file 1 [file animals-11-02081-s001.zip › Supplementary data/Table S1.pdf]

	Baseline	30	60	90	120
Cardiac index (mL/kg/min) Reference range: 72-88 mL/kg/min					
M	58.1±13.8	43.9±11.1	48.4±16.9	44.5±18.0	46.4±15.3
MK	75.1±27.8	64.4±24.4	50.8±15.5	52.4±11.6	51.4±20.2
MMo	68.8±31.9	43.9±8.9	41.8±13.2*	43.8±16.2	41.0±12.9*
MMoK	66.9±20.2	52.6±18.7	52.2±18.7	50.6±15.6	58.7±17.3

Table S1. Doppler echocardiographic measurement of cardiac output at the pulmonary artery. * Significantly different from baseline within a treatment.
