# Supplementary material for: Cardiorespiratory, Sedative and Antinociceptive Effects of a Medetomidine Constant Rate Infusion with Morphine, Ketamine or Both
Source: Animals (Basel). 2021 Jul 13;11(7):2081. doi: 10.3390/ani11072081 (PMC8300393; doi:10.3390/ani11072081)
Supplement: Supplementary file 1 [file animals-11-02081-s001.zip › Supplementary data/Table S2.pdf]

	Baseline	Bolus (-5)	60	120
PR interval (ms) Reference range: ≤ 500 ms				
M	299.4±77.2	313.0±43.2	315.3±78.9	317.0±52.3
MK	279.4±33.5	297.6±43.4	297.9±38.9	293.6±54.7
MMo	284.0±16.5	299.3±32.6	284.6±44	278.8±48.3
MMoK	279.0±56.4	320.2±51.3	320.2±51.3	292.0±41.8
QS interval (ms) Reference range: ≤140 ms				
M	106.0±8.0	116.1±15.4	112.2±14.1	113.2±13.2
MK	104.6±4.6	105.0±4.5	104.7±8.3	104.3±12.6
MMo	101.9±5.7	107.2±3.9	103.4±6.6	100.8±8.7
MMoK	105.1±11.6	106.8±14.3	100.8±8.7	102.9±9.3
QT interval (ms) Reference range: ≤ 600 ms				
M	512.8±29.0	504.0±44.0	569.3±30.3*	597.0±16.5*
MK	505.2±36.2	497.2±56.4	569.3±45.8*	595.3±51.0*
MMo	504.2±47.8	510.0±29.1	582.2±43.3*	611.1±44.8*
MMoK	532.7±30.9	517.0±43.6	584.9±46.4*	603.2±63.4*
QTc				
M	14.0±1.3	18.6±5.4	18.2±4.2	20.0±3.7
MK	12.0±3.2	14.7±2.6	17.1±3.4	18.9±2.4*
MMo	12.9±2.4	19.7±8.5*	19.6±3.9*	19.0±3.3*
MMoK	14.4±1.9	17.3±0.9	19.8±5.1	19.0±2.6

Table S2. Electrocardiographic intervals duration. * Significantly different from baseline within a treatment.
