# Supplementary material for: Cardiorespiratory, Sedative and Antinociceptive Effects of a Medetomidine Constant Rate Infusion with Morphine, Ketamine or Both
Source: Animals (Basel). 2021 Jul 13;11(7):2081. doi: 10.3390/ani11072081 (PMC8300393; doi:10.3390/ani11072081)
Supplement: Supplementary file 1 [file animals-11-02081-s001.zip › Supplementary data/Table S3.pdf]

	Baseline	30	60	120
pH (ref. 7.35-7.45)				
M	7.452±0.085	7.436±0.072	7.449±0.058	7,444±0.058[a]
MK	7.342±0.194	7.430±0.034	7.420±0.023	7.432±0.035
MMo	7.450±0.045	7.440±0.036	**7.462±0.048**	**7.487±0.037**
MMoK	7.451±0.059	7.440±0.054	7.429±0.049	7.392±0.132[a]
$PaCO_2$ mmHg (ref. 35-45)				
M	39.9±7.5	44.4±4.0	45.0±4.6	**48.5±6.3**
MK	43.9±4.2	45.7±2.9	**49.0±3.9**	**50.2±4.9**
MMo	44.1±3.4	**49.4±4.3**	**49.1±7.0**	**46.8±4.7**
MMoK	41.7±4.7	45.9±5.3	**50.4±4.8**	**47.2±4.0**
PaO_2 mmHg (ref. 73-98)				
M	91.9±17.0	73.0±12.4	81.0±8.4	83.9±9.6
MK	80.8±7.8	83.6±5.5	78.5±11.7	80.1±4.4
MMo	78.6±5.7	**68.3±7.5**	80.2±18.5	81.8±8.8
MMoK	85.3±10.5	78.9±10.5	76.4±3.5	79.4±7.4
Na mmol/L (ref. 135-145)				
M	136.2±3.5	139.4±6.2	138.8±5.6	139.5±9.0
MK	141.5±5.8	138.9±3.3	138.5±3.4	138.8±3.4
MMo	137.2±1.5	140.6±3.3	139.7±2.8	137.3±3.9
MMoK	137.9±5.5	136.7±3.6	138.3±8.0	138.3±4.5
K mmol/L (ref. 3.5-4.5)				
M	4.27±0.44	4.33±0.54	4.36±0.49	4.37±0.65
MK	4.20±0.44	4.17±0.55	4.30±0.67	4.47±0.80
MMo	4.13±0.31	4.32±0.35	4.45±0.53	4.63±0.76*
MMoK	4.13±0.38	4.07±0.36	4.19±0.42	4.29±0.53
iCa mmol/L (ref. 1.4-1.6)				
M	1.65±0.13[a,b]	1.60±0.16[c]	1.56±0.17[d]	1.52±0.20*[,e]
MK	1.49±0.25[a]	1.43±0.19[c]	1.43±0.20[d]	1.41±0.16
MMo	1.50±0.17[b]	1.47±0.18	1.48±0.18	1.40±0.16
MMoK	1.58±0.16	1.50±0.13	1.51±0.29	1.46±0.17*[,e]
HCO_3 mmol/L (ref. 25-32)				
M	27.8±6.1[a]	30.2±6.5*[,d]	31.3±6.1*[,g]	33.3±7.1*
MK	28.5±3.8[b]	30.0±3.8[e]	31.4±3.3*[,h]	32.8±2.4*
MMo	30.6±2.9[a,b,c]	33.2±3.6*[,d,e,f]	34.3±3.0*[,g,h]	34.8±2.2*
MMoK	28.2±1.2[c]	30.9±1.6*[,f]	32.3±1.9*	33.1±1.3*
SBE mmol/L (ref. -3 to +3)				
M	3.6±6.1	5.2±6.8	6.4±6.1*	7.7±6.8*
MK	1.8±5.3[a]	4.9±3.8*[,b]	5.7±2.9*[,c]	7.2±2.3*[,d]
MMo	5.9±3.1[a]	7.6±3.3[b]	8.9±2.5*[,c]	9.9±2.0*[,d]
MMoK	4.1±2.0	5.9±2.2	6.6±2.3	7.5±1.6*

SO$_2$ % (ref. > 95)				
M	96.7±1.5[a]	**94.3±2.7**	96.2±0.8	96.4±1.0
MK	**92.4±5.7**[a]	96.1±1.2	95.4±1.3	95.9±0.7
MMo	96.0±0.5	**93.9±1.7**	95.4±3.0	96.5±1.0
MMoK	96.5±1.2	95.7±1.0	95.4±1.0	96.0±1.1

Table S3. Partial gas arterial pressures, electrolytes and acid-base parameters. * Significantly different from baseline within a treatment. abcd Different superscript letters indicate significant differences between treatments at this timepoint. Bold numbers indicate values above or below reference range.
