# Supplementary material for: Cardiorespiratory, Sedative and Antinociceptive Effects of a Medetomidine Constant Rate Infusion with Morphine, Ketamine or Both
Source: Animals (Basel). 2021 Jul 13;11(7):2081. doi: 10.3390/ani11072081 (PMC8300393; doi:10.3390/ani11072081)
Supplement: Supplementary file 1 [file animals-11-02081-s001.zip › Supplementary data/Table S4.pdf]

	Baseline	0	10	20	30	40	50	60	70	80	90	100	110	120	Median
M	100	44±15*	54±17*	51±17*	53±18*	50±16*	46±17*	38±14*	46±13*	39±11*	40±16*	39±15*	39±13*	43±15*	45±6
MK	100	63±16*	64±14*	67±22*	59±8*	61±16*	59±14*	56±10*	55±11*	54±6*	58±5*	56±12*	53±8*	56±11*	59±4
MMo	100	58±17*	61±15*	59±13*	58±14*	57±17*	57±15*	57±11*	51±12*	50±9*	55±20*	54±13*	55±13*	51±*13	56±3
MMoK	100	45±14*	50±11*	48±12*	45±12*	50±10*	46±13*	45±8*	52±12*	48±12*	51±16*	52±14*	47±14*	49±15*	48±2

Table S4. Percentage of head height to ground with baseline as reference (100%). * Significantly different from baseline within a treatment.
