# Supplementary material for: Cardiorespiratory, Sedative and Antinociceptive Effects of a Medetomidine Constant Rate Infusion with Morphine, Ketamine or Both
Source: Animals (Basel). 2021 Jul 13;11(7):2081. doi: 10.3390/ani11072081 (PMC8300393; doi:10.3390/ani11072081)
Supplement: Supplementary file 1 [file animals-11-02081-s001.zip › Supplementary data/Table S5.pdf]

	0	10	20	30	40	50	60	70	80	90	100	110	120
M	0.09±0.22	0.02±0.04	0±0	0.03±0.08	0.02±0.04	0.02±0.04	0±0	0±0	0±0	0±0	0±0	0±0	0±0
MK	0.28±0.56[a]	0.03±0.06*	0.05±0.08*	0.04±0.04*	0.05±0.08*	0.04±0.04*	0±0*	0.02±0.03*	0.01±0.02*	0.03±0.08	0±0*	0±0*	0.04±0.10*
MMo	0.12±0.22	0.21±0.49	0.04±0.10	0.01±0.02	0.01±0.02	0.03±0.06	0.03±0.06	0.01±0.02	0±0	0.01±0.02	0±0	0±0	0±0
MMoK	0.03±0.06[a]	0±0	0±0	0±0	0±0	0±0	0±0	0±0	0±0	0±0	0±0	0±0	0±0

Tables S5. Visual Analogic Scale evaluation of ataxia (cm) between 0 (normal horse) to 10 (horse unable to stand). * Significantly different from baseline within a treatment. abcd Different superscript letters indicate significant differences between treatments at this timepoint.
